# Optimal message-passing with noisy beeps

**DOI:** 10.1007/s00446-025-00488-6

**Published:** 2025-06-10

**Authors:** Peter Davies-Peck

**Affiliations:** Computer Science, Durham University, Durham, DH1 3LE United Kingdom

**Keywords:** Message Passing, Beeping Model, Superimposed Codes

## Abstract

Beeping models are models for networks of weak devices, such as sensor networks or biological networks. In these networks, nodes are allowed to communicate only via emitting beeps: unary pulses of energy. Listening nodes have only the capability of *carrier sensing*: they can only distinguish between the presence or absence of a beep, but receive no other information. The noisy beeping model further assumes listening nodes may be disrupted by random noise. Despite this extremely restrictive communication model, it transpires that complex distributed tasks can still be performed by such networks. In this paper we provide an optimal procedure for simulating general message passing in the beeping and noisy beeping models. We show that a round of Broadcast CONGEST can be simulated in $O(\Delta \log n)$ rounds of the noisy (or noiseless) beeping model, and a round of CONGEST can be simulated in $O(\Delta ^2\log n)$ rounds (where $\Delta$ is the maximum degree of the network). We also prove lower bounds demonstrating that no simulation can use asymptotically fewer rounds. This allows a host of graph algorithms to be efficiently implemented in beeping models. We present several example applications, including an $O(\log n)$-round Broadcast CONGEST algorithm for maximal matching, which, when simulated using our method, immediately implies a near-optimal $O(\Delta \log ^2 n)$-round maximal matching algorithm in the noisy beeping model. A preliminary version of this paper appeared in the proceedings of the 2023 ACM Symposium on Principles of Distributed Computing (PODC) [14].

## Introduction

Beeping models were first introduced by Cornejo and Kuhn [11] to model wireless networks of weak devices, such as sensor networks and biological networks [2]. These models are characterised by their very weak assumptions of communication capabilities: devices are assumed to communicate only via *carrier sensing*. That is, they have the ability to distinguish between the presence or absence of a signal, but not to gain any more information from the signal.

### Models

The models we study all have the same basic structure: a network of devices is modeled as a graph with *n* nodes (representing the devices) and maximum degree $\Delta$, where edges represent direct reachability between pairs of devices. We will assume that all nodes activate simultaneously, and therefore have shared global clock (some prior work on beeping models instead allow nodes to activate asynchronously). Time then proceeds in synchronous rounds, in which nodes can perform some local computation and then can communicate with neighboring devices. The defining characteristic of each model is the communication capability of the nodes.

***Noiseless Beeping Model***

In each round, each node chooses to either beep or listen. Listening nodes then hear a beep iff at least one of their neighbors beeped, and silence otherwise. Nodes do not receive any other information about the number or identities of their beeping neighbors.

***Noisy Beeping Model***

The noisy beeping model, introduced by Ashkenazi, Gelles, and Leshem [4], is similar to the noiseless version, except that the signal each listening node hears (beep or silence) is *flipped*, independently uniformly at random, with some probability $\varepsilon \in (0,\frac{1}{2})$.

Within these beeping models, our aim will be to simulate more powerful message-passing models, in which nodes have the ability to send longer messages to each other, and these messages are received without interference:

Broadcast CONGEST ***Model***

In rounds of the Broadcast CONGEST model, nodes may send the same $O(\log n)$-bit message to each of their neighboring nodes, and each node hears the messages from all of its neighbors.

CONGEST ***Model***

The CONGEST model is similar to Broadcast CONGEST, but allows nodes to send (potentially) different $O(\log n)$-bit messages to each of their neighboring nodes. Again, each node hears the messages from all of its neighbors.

The communication capabilities in the Broadcast CONGEST and CONGEST models are clearly much more powerful than that of either beeping model, and CONGEST in particular has a broad literature of efficient algorithms. Our aim in this work is to provide an efficient generic simulation of Broadcast CONGEST and CONGEST in the beeping models, so that these existing algorithms can be applied out-of-the-box to networks of weak devices.

### Prior work

***Beeping models***

The (noiseless) beeping model was introduced by Cornejo and Kuhn [11], who also gave results for an interval coloring task used for synchronization. Classical local graph problems have been studied in the model, with Afek et al. [1] giving an $O(\log ^2 n)$-round maximal independent set algorithm, and Beauqier et al. [7] giving $O(\Delta ^2 \log n + \Delta ^3)$-round deterministic algorithms for maximal independent set and $(\Delta +1)$-coloring.

Global communication problems (those requiring coordination across the entire network, and therefore with running times parameterized by the diameter *D* of the network) have also been studied. Single-source broadcast of a *b*-bit message can be performed in $O(D+b)$ rounds using the simple tool of ‘beep waves’, introduced by Ghaffari and Haeupler [22] and formalized by Czumaj and Davies [12]. Leader election, another fundamental global problem, has seen significant study in the model. Ghaffari and Haeupler [22] gave a randomized algorithm requiring $O(D+\log n \log \log n)\cdot \min \{\log \log n, \log \frac{n}{D}\}$ rounds, while Förster, Seidel and Wattenhofer [19] gave an $O(D \log n)$-round *deterministic* algorithm. Czumaj and Davies [13] gave a simple randomized algorithm with $O(D \log n)$ worst-case round complexity but $O(D+\log n)$ expected complexity. Finally, Dufoulon, Burman and Beauquier [15] settled the complexity of the problem with a deterministic algorithm with optimal $O(D+\log n)$ round complexity.

On other global problems, Czumaj and Davies [12] and Beauqier et al. [6] gave results for broadcasting from multiple sources, and Dufoulon, Burman and Beauqier [16] study synchronization primitives for the model variant where nodes activate asynchronously.

***Message passing models***

Message passing models, and CONGEST in particular, have seen a long history of study and have a rich literature of algorithms for problems including (among many others) local problems such as $\Delta +1$-coloring[23], global problems such as minimum spanning tree[27], and approximation problems such as approximate maximum matching[3]. Broadcast CONGEST is less well-studied, though some dedicated algorithms have also been developed for it, e.g. [10]. There is an obvious way to simulate CONGEST algorithms in Broadcast CONGEST at an $O(\Delta )$-factor overhead: nodes simply broadcast the messages for each of their neighbors in turn, appending the ID of the intended recipient. In general this is the best that can be done (as can be seen from our bounds on simulating beeping models), but for specific problems this $\Theta (\Delta )$ complexity gap is often not necessary.

***Simulating message passing with beeps***

Two works have previously addressed the task of simulating message passing in beeping models. The first was by Beauquier et al. [7], and gave a generic simulation for CONGEST in the noiseless beeping model. Their algorithm required $\Delta ^6$ setup rounds, and then $\Delta ^4\log n$ beep-model rounds per round of CONGEST. This result was improved by Ashkenazi, Gelles, and Leshem [4], who introduced the noisy beeping model, and gave an improved simulation of CONGEST which requires $O(\Delta ^4\log n)$ rounds of setup, and then simulates each CONGEST round in $O(\Delta \log n \cdot \min \{n,\Delta ^2\})$ rounds of noisy beeps.

### Our results

We give a randomized simulation of Broadcast CONGEST which requires $O(\Delta \log n)$ rounds in the noisy beep model per round of Broadcast CONGEST, with no additional setup cost. We will call this per-round cost the *overhead* of simulation. This implies a simulation of CONGEST with $O(\Delta ^2\log n)$ overhead in the noisy beep model. We therefore improve over the previous best result of [4] by reducing the overhead by a $\Theta (\min \{\frac{n}{\Delta },\Delta \})$ factor, and removing the large setup cost entirely. We prove that these bounds are tight for both Broadcast CONGEST and CONGEST by giving matching lower bounds (even for the noiseless beeping model). This has the potentially surprising implication that introducing noise into the beeping model does not asymptotically increase the complexity of message-passing simulation at all.

This simulation result allows many CONGEST and Broadcast CONGEST algorithms to be efficiently implemented with beeps. We give several example applications, including an $O(\log n)$-round Broadcast CONGEST algorithm for the task of maximal matching, which via our simulation implies an $O(\Delta \log ^2 n)$-round algorithm in the noisy beeping model. We show that this is almost optimal by demonstrating an $\Omega (\Delta \log n)$ lower bound (even in the noiseless model).

### Our approach

We summarize our approach to simulating CONGEST in the noiseless beeping model (the noisy case will follow naturally, as we will see later). First, let us mention the general approach of the previous results of [7] and [4]: there, the authors use a coloring of $G^2$ (i.e., a coloring such that no nodes within distance 2 in *G* receive the same color) to sequence transmissions. They iterate through the color classes, with nodes in each class transmitting their message (over a series of rounds, with a beep or silence representing each bit of the message). Since nodes have at most one neighbor in each color class, they hear that neighbor’s message undisrupted.

The disadvantage of such an approach is that the coloring of $G^2$ requires a large setup time to compute, and also necessitates at least $\min \{n,\Delta ^2\}$ color classes. This is the cause of the larger overhead in the simulation result of [4].

Instead of having nodes transmitting at different times, our solution is to have them all transmit at once, and use superimposed codes to ensure that the messages are decipherable. The definition of a classic superimposed code is as follows:

#### Definition 1

(Superimposed Codes) An (*a*, *k*)-superimposed code of length *b* is a function $C:\{0,1\}^a\rightarrow \{0,1\}^b$ such that any superimposition (bitwise OR) of at most *k* codewords is unique.

The connection between superimposed codes and beeping networks is that, if some subset of a node *v*’s neighbors all transmit a message simultaneously (using beeps to represent ${\textbf {1}}$s and silence to represent ${\textbf {0}}$s), then *v* (if it were to listen every round) would hear the bitwise OR superimposition of all the messages. If this superimposition is unique, then *v* is able to identify the set of messages that were transmitted (and this set contain precisely those messages with no ${\textbf {1}}$ in a position where the superimposition has ${\textbf {0}}$).

Superimposed codes of this form were first introduced by Kautz and Singleton [26], who showed a construction with $b=O(k^2 a)$. This definition is equivalent to cover-free families of sets, which is the terminology used in much of the prior work. A lower bound $b=\Omega (\frac{k^2 a}{\log k} )$ was found by D’yachkov and Rykov [17], with a combinatorial proof later given by Ruszinkó [31], and another, simple proof given by Füredi [20]. The $\log k$ gap between upper and lower bounds remains open.

This presents a problem to applying such codes for message passing in the beep model. If all nodes are transmitting their message (of $O(\log n)$ bits) at once, then we would need to use an $(O(\log n),\Delta )$-superimposed code for the messages to be decodable. Using Kautz and Singleton’s construction [26] results in a length of $O(\Delta ^2\log n)$ (and length corresponds directly to rounds in the beeping model). This would result in the same $O(\Delta ^2)$-factor overhead as from using a coloring of $G^2$, so would not improve over [4]. Furthermore, even if we were to find improved superimposed codes, the lower bound implies that any such improvement would be only minor.

To achieve codes with better length, we weaken the condition we require. Rather than requiring that all superimpositions of at most *k* codewords are unique, we only require that *most* are. Specifically, if the *k* codewords are chosen at random, then their superimposition will be unique (and hence decodable) with high probability. We show the existence of short codes with this weakened property. Constructions with similar properties (though not quite suitable for our uses) were also given in [18].

This raises a new problem: using these shorter codes, we can efficiently have all nodes send a *random* message to their neighbors, but how does this help us send a specific message?

Our answer is that if were to repeat the transmission (using the same random codewords for each node), then every node *v* would already know exactly when its neighbors should be beeping^1^, and in particular, *v* knows when a neighbor *u* should be beeping *alone* (i.e., not at the same time as any other neighbor of *v*). If *u* now beeps only in a *subset* of the rounds indicated by its codeword, then it can pass information to *v* in this way. So, our final algorithm uses a secondary *distance* code to specify what this subset should be in order to ensure that all neighbors of *u* can determine *u*’s message. The aim of this distance code is that codewords are sufficiently large Hamming distance apart that *u*’s neighbors can determine *u*’s message, even though they only hear a subset of the relevant bits, and these bits can be flipped by noise in the noisy model.

### Notation

Our protocols will be heavily based on particular types of binary codes, which we will communicate in the beeping model via beeps and silence. In a particular round, in the noiseless beeping model, we will say that a node *v* receives a $\textbf{1}$ if it either listens and hears a beep, or beeps itself. We will say that *v* receives a $\textbf{0}$ otherwise. In the noisy model, what *v* hears will be this bit, flipped with probability $\varepsilon$.

We will use logic operators to denote operations between two strings: for $s,s'\in \{0,1\}^a$, $s\wedge s'\in \{0,1\}^a$ is the logical And of the two strings, with $\textbf{1}$ in each coordinate iff both *s* and $s'$ had $\textbf{1}$ in that coordinate. Similarly, $s\vee s'\in \{0,1\}^a$ is the logical Or of the two strings, with $\textbf{1}$ in each coordinate iff *s* or $s'$ (or both) had $\textbf{1}$ in that coordinate.

#### Definition 2

We will use $\textbf{1}(s)$ to denote the number of $\textbf{1}$s in a string $s\in \{0,1\}^a$. We will say that a string $s\in \{0,1\}^a$ *d*-intersects another string $s'\in \{0,1\}^a$ if $\textbf{1}(s\wedge s')\ge d$.

For a set of strings $S\in \{0,1\}^a$, we will use $\vee (S)$ as shorthand for the superimposition $\bigvee _{s\in S}s$.

Finally, for a node *v* in a graph $G=(V,E)$, we will use *N*(*v*) to denote the *inclusive* neighborhood of *v*, i.e. $\{u\in V: \{u,v\}\in E\} \cup \{v\}$.

## Binary codes

The novel type of superimposed code on which our algorithm is mainly based is defined as follows:

### Definition 3

An $(a,k,\delta )$-beep code of length *b* is a function $C:\{0,1\}^a\rightarrow \{0,1\}^b$ such that:

- all $$s\in C$$ have $$\textbf{1}(s)=\frac{\delta b}{k}$$.
- the number of size-*k* subsets $S\subseteq C$ whose superimpositions $\vee (S)$ $\frac{5\delta ^2 b}{k}$-intersect some $s\in C\setminus S$ is at most $$\left( {\begin{array}{c}2^a\\ k\end{array}}\right) 2^{-2a}$$

(here we slightly abuse notation by using *C* to denote the set of codewords, i.e. the image $C(\{0,1\}^a)$ of the beep code function).

In other words, all codewords have exactly $\frac{\delta b}{k}$ $\textbf{1}$s, and only a $2^{-2a}$-fraction of the $$\left( {\begin{array}{c}2^a\\ k\end{array}}\right) $$ size-*k* subsets of codewords have a superimposition that $\frac{5\delta ^2 b}{k}$-intersects some other codeword. This first criterion is only a technicality to aid our subsequent application; the important point is the second, which, we will show, implies that a superimposition of *k* *random* codewords will, with probability at least $1-2^{-2a}$, be decodable (even under noise, since avoiding $\frac{5\delta ^2 b}{k}$-intersection will provide us with sufficient redundancy to be robust to noise). Note that for such a code to exist, $\frac{\delta b}{k}$ must be an integer, which we will guarantee in our construction.

### Theorem 1

For any $a,k,c\in \mathbb {N}$, there exists an (*a*, *k*, 1/*c*)-beep code of length $b=c^2 ka$.

### Proof

The proof will be by the probabilistic method: we will randomly generate a candidate code *C*, and then prove that it has the desired properties with high probability in $2^a$. Then, a code with such properties must exist, and the random generation process we use implies an efficient algorithm to find such a code with high probability (though *checking* the code is correct would require $2^{O(ak)}$ computation).

To generate our candidate code, we choose each codeword independently, uniformly at random from the set of all *b*-bit strings with $\frac{ b}{ck}$ $\textbf{1}$s. This clearly guarantees the first property.

For a fixed size-*k* set *S* of codewords, and a fixed codeword $x\in C\setminus S$, we now analyze the probability that $\vee (S)$ $\frac{5 b}{c^2k}$-intersects *x*.

Clearly we have $\textbf{1}(\vee (S)) \le k\cdot \frac{ b}{ck} = b/c$. Consider the process of randomly choosing the positions of the **1**s of *x*. The probability that any particular fixed set of $\frac{5 b}{c^2k}$ positions receives all **1**s in *x* is

$$\begin{aligned}\frac{\frac{b}{ck}}{b} \cdot \frac{\frac{b}{ck}-1}{b -1} \dots \frac{\frac{b}{ck}-\frac{5 b}{c^2k}}{b -\frac{5 b}{c^2k}} < \left( {ck}\right) ^{-\frac{5 b}{c^2k}}. \end{aligned}$$

Taking a union bound over all possible size-$\frac{5 b}{c^2k}$ subsets of positions where $\vee (S)$ has **1**s, the probability that $\vee (S)$ $\frac{5 b}{c^2k}$-intersects *x* is therefore at most

$$\begin{aligned} \left( {\begin{array}{c}\frac{b}{c}\\ \frac{5 b}{c^2k}\end{array}}\right) \cdot (ck)^{-\frac{5 b}{c^2k}}&\le \left( \frac{eck}{5}\right) ^{\frac{5 b}{c^2k}}\cdot (ck)^{-\frac{5 b}{c^2k}}\\&\le \left( \frac{5}{e}\right) ^{-\frac{5 c^2ka}{c^2k}} \le 2^{-4 a}\hspace{5.0pt}.\end{aligned}$$

Taking a union bound over all codewords $s\in C\setminus S$, we find that the probability that $\vee (S)$ $\frac{5 b}{c^2k}$-intersects any such codeword is at most $2^{-3a}$. Then, the expected number of size-*k* sets *S* that $\frac{5 b}{c^2k}$-intersect any $s\in C\setminus S$ is at most $$\left( {\begin{array}{c}2^a\\ k\end{array}}\right) 2^{-3a}$$. By the probabilistic method, there therefore *exists* a an (*a*, *k*, 1/*c*)-beep code in which the number of size-*k* sets *S* that $\frac{5 b}{c^2k}$-intersect any $s\in C\setminus S$ is at most $$\left( {\begin{array}{c}2^a\\ k\end{array}}\right) 2^{-3a}$$.

However, since we also want an efficient algorithm to *find* an (*a*, *k*, 1/*c*)-beep code, we note that by Markov’s inequality the probability that more than $$\left( {\begin{array}{c}2^a\\ k\end{array}}\right) 2^{-2a} $$ size-*k* sets *S* that $\frac{5 b}{c^2k}$-intersect any $s\in C\setminus S$ is at most $2^{-a}$, and therefore the process of choosing codewords uniformly at random from all strings with $\frac{ b}{ck}$ $\textbf{1}$s gives an (*a*, *k*, 1/*c*)-beep code with probability at least $1-2^{-a}$.

$$\square $$

Notice that, while the theorem holds for any $c\in \mathbb {N}$, it is trivial for $c\le 2$: in this case, codewords cannot $\frac{5 b}{c^2k}$-intersect any string, since they contain only $\frac{b}{ck}$ **1**s. Our application will set *c* to be a sufficiently large constant.

Our algorithm will also make use of *distance codes*. These codes have the simple criterion that every pair of codewords is sufficiently far apart by Hamming distance (which we will denote $d_H$). Distance codes are an example of error-correcting codes, which have a wealth of prior research (see e.g. [25] for an extensive survey); here we just require a very simple object, for which we give a proof in a similar style to that of Theorem 1 for consistency:

### Definition 4

An $(a,\delta )$-distance code of length *b* is a function $D:\{0,1\}^a\rightarrow \{0,1\}^b$ such that all pairs $s\ne s'\in D$ have $d_H(s,s')\ge \delta b$.

### Lemma 2

For any $\delta \in (0,\frac{1}{2})$, $a\in \mathbb {N}$, and $c_\delta \ge 12(1-2\delta )^{-2}$, there exists an $(a,\delta )$-distance code of length $b=c_\delta a$.

### Proof

We randomly generate a candidate code by choosing each codeword’s entries independently uniformly at random from $\{0,1\}$. For any pair of codewords $s,s'\in D$, the probability that they differ on any particular entry is $\frac{1}{2}$. The expected distance is therefore $\frac{b}{2}$, and by a Chernoff bound,

$$\begin{aligned}&\textbf{Pr}\left[ d_H(s,s') \le \delta b\right] = \textbf{Pr}\left[ d_H(s,s')\le 2\delta \textbf{E}\left[ d_H(s,s')\right] \right] \\&\hspace{1cm}\le e^{\frac{-(1-2\delta )^2\textbf{E}\left[ d_H(s,s')\right] }{2}} = e^{\frac{-(1-2\delta )^2c_\delta a}{4}}\hspace{5.0pt}. \end{aligned}$$

Since $$c_\delta \ge 12(1-2\delta )^{-2}$$,

$$\begin{aligned} \textbf{Pr}\left[ dist(s,s') \le \delta b\right]&\le e^{-3 a}\le 2^{-4a}\hspace{5.0pt}. \end{aligned}$$

Taking a union bound over all $$\left( {\begin{array}{c}2^a\\ 2\end{array}}\right) \le 2^{2a}$$ pairs $s,s'\in D$, we find that the probability that any pair has $dist(s,s')\le \delta b$ is at most $2^{-2a}$. Therefore, the random generation process generates an $(a,\delta )$-distance code with probability at least $1-2^{-2a}$. $\square$

This construction can also be checked relatively efficiently, since one need only check the distance of $O(2^{2a})$ codeword pairs, which can be performed in $2^{O(a)}$ computation.

## Simulation algorithm

We now arrive at our main simulation algorithm. We give an algorithm for simulating a single communication round in Broadcast CONGEST using $O(\Delta \log n)$ rounds of the noisy beep model. What we mean by this simulation is that each node *v* begins with a $\gamma \log n$-bit message $m_v$ to transmit to all neighbors (where $\gamma$ is some constant, a parameter of the Broadcast CONGEST model), and by the end of our beeping procedure, all nodes should be able to output the messages of all their neighbors.

Let $c_\varepsilon$ be a constant to be chosen based on $\varepsilon$, the noise constant. Our algorithm will make use of two codes (instantiations of those defined in the previous section):

- a $(\gamma \log n,\frac{1}{3})$-distance code *D* of length $c_\varepsilon ^2 \gamma \log n$, given by Lemma 2 (so long as we choose $c_\varepsilon \ge 108$);
- a $(c_\varepsilon \gamma \log n,\Delta +1,1/c_\varepsilon )$-beep code *C* of length $c_\varepsilon ^3 \gamma (\Delta +1)\log n$ given by Theorem 1 .

The codewords in the beep code *C* contain exactly $c_\varepsilon ^2 \gamma \log n$ $\textbf{1}$s. The purpose of using these two codes is to combine them in the following manner:

### Notation 3

For a binary string *s*, let ${\textbf {1}}_i(s)$ denote the position of the $i^{th}$ ${\textbf {1}}$ in *s* (and Null if *s* contains fewer than *i* ${\textbf {1}}$s).

Let $CD:\{0,1\}^{c_\varepsilon \gamma \log n}\times \{0,1\}^{\gamma \log n} \rightarrow \{0,1\}^{c_\varepsilon ^3 \gamma (\Delta +1)\log n}$ be the combined code defined as follows:

$$\begin{aligned} CD(r,m)_j = {\left\{ \begin{array}{ll} \textbf{1} & \begin{array}{c} \text { if } \exists i\in [c_\varepsilon ^2 \gamma \log n], {\textbf {1}}_i(C(r)) = j,\\ \text {and } D(m)_i = \textbf{1} \end{array} \\ \textbf{0 }&  \text {otherwise}\end{array}\right. }\end{aligned}$$

**Fig. 1:**
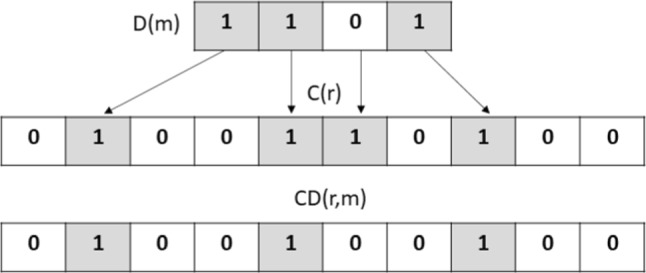
Combined code construction

That is, *CD*(*r*, *m*) is the code given by writing the codeword *D*(*m*) in the positions where *C*(*r*) is ${\textbf {1}}$ (and leaving the other positions as ${\textbf {0}}$): see Figure 1.

The algorithm is then as follows (Algorithm 1):

**Algorithm 1:**
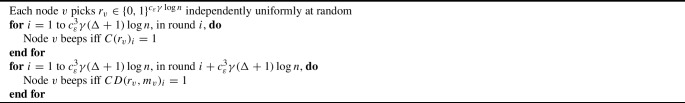
Simulation of a Broadcast CONGEST round in the noisy beeping model

So, each node picks a random codeword from the beep code, and transmits it bitwise using beeps and silence. By the properties of the beep code, with high probability the superimposition of messages each node receives will be decodable. Then, to actually convey the message $m_v$, *v* uses the combined code, which transmits $m_v$, encoded with a distance code, in the positions where the beep codeword $r_v$ used in the first round was ${\textbf {1}}$. Neighbors *u* of *v* know when these positions are from the first round. Of course, there are some rounds when other neighbors of *u* will be beeping, some rounds when *u* must beep itself and cannot listen, and some rounds when the signal from *v* is flipped by noise. However, we will show that, by a combination of the properties of our two codes, there is sufficient redundancy to overcome all three of these obstacles, and allow *u* to correctly decode *v*’s message.

## Decoding the code

In the first phase, each node *v* hears^2^ a string we will denote $\tilde{x}_v$, which is the string $x_v:= \bigvee _{u\in N(v)} C(r_u)$ with each bit flipped with probability $\varepsilon \in (0,\frac{1}{2})$, and the aim is for *v* to decode this string in order to determine the set $R_v:= \{r_u:u\in N(v)\}$.

We first show that, before considering noise, with high probability the superimposition of random codewords chosen by each node’s inclusive neighborhood is decodable.

### Lemma 4

With probability at least $1- n^{3-c_\varepsilon \gamma }$, for every node $v\in V$ and every $r\in \{0,1\}^{c_\varepsilon \gamma \log n}$, *C*(*r*) does not $5c_\varepsilon \gamma \log n$-intersect $\bigvee _{r_u\in R_v\setminus \{r\}} C(r_u)$.

### Proof

First, we see that with probability at least $1-\frac{n^2}{2^{c_\varepsilon \gamma \log n}} = 1-n^{2-c_\varepsilon \gamma }$, all nodes choose different random strings. For the rest of the proof we condition on this event.

For each $v\in V$, $r\in \{0,1\}^{c_\varepsilon \gamma \log n}$, let $R_{v,r}$ be a set of nodes’ random strings defined as follows: starting with $R_v\setminus \{r\}$ (which is a set of input messages of size at most $\Delta +1$), add arbitrary $r_x$ from nodes $x\notin (N(v)\cup \{r\})$ until the set is of size exactly $\Delta +1$. Since we are conditioning on the event that all nodes generate different random strings, $R_{v,r}$ is a set of $\Delta +1$ distinct random strings from $\Delta +1$ distinct nodes, none of which are *r*.

By the properties of a $(c_\varepsilon \gamma \log n,\Delta +1,1/c_\varepsilon )$-beep code, therefore, the probability that *C*(*r*) $5c_\varepsilon \gamma \log n$-intersects $\bigvee _{r_u\in R_{v,r}} C(r_u)$ is at most $2^{-2c_\varepsilon \gamma \log n} = n^{-2c_\varepsilon \gamma }$. If *C*(*r*) does not $5c_\varepsilon \gamma \log n$-intersect $\bigvee _{r_u\in R_{v,w}} C(r_u)$, then since $R_{v,r}$ is a superset of $R_v\setminus \{r\}$, *C*(*r*) also does not $5c_\varepsilon \gamma \log n$-intersect $\bigvee _{r_u\in R_v\setminus \{r\}} C(r_u)\hspace{5.0pt}$.

The number of possible pairs $v\in V$, $r\in \{0,1\}^{c_\varepsilon \gamma \log n}$ is $n^{1+c_\varepsilon \gamma }$. Taking a union bound over all of these, we find that *C*(*r*) does not $5c_\varepsilon \gamma \log n$-intersect $\bigvee _{r_u\in R_v\setminus \{r\}} C(r_u)$ for any pair with probability at least $1-n^{1+c_\varepsilon \gamma -2c_\varepsilon \gamma } = 1-n^{1-c_\varepsilon \gamma }$ by a union bound. Finally, removing the conditioning on the event that nodes’ random strings are all different, we reach the condition of the lemma with probability at least $1-n^{1-c_\varepsilon \gamma } - n^{2-c_\varepsilon \gamma } \ge 1- n^{3-c_\varepsilon \gamma }$. $\square$

Next we must analyze how noise affects the bitstrings that nodes hear. For any node *v*, let $x_v$ denote the string *v* heard, i.e., $\bigvee _{u\in N(v)} C(r_u)$, after each bit is flipped with probability $\varepsilon \in (0,\frac{1}{2})$. To decode the set $R_v$, *v* will take

$$\begin{aligned}&\tilde{R}_v = \{r\in \{0,1\}^{c_\varepsilon \gamma \log n}\text { such that:}\\&C(r) \text { does not } \frac{2\varepsilon +1}{4}c_\varepsilon ^2\gamma \log n \text { -intersect }\lnot \tilde{x}_v\}\hspace{5.0pt}. \end{aligned}$$

That is, it includes all codewords which have fewer than $\frac{2\varepsilon +1}{4}c _\varepsilon ^2\gamma \log n$ **1**s in positions where $\tilde{x}_v$ does not.

Notice that, in the absence of noise, all *C*(*r*) for $r\in R_v$ have zero **1**s in positions where $x_v$ did not, and all *C*(*r*) for $r\notin R_v$ have at least $c_\varepsilon (c_\varepsilon - 5)\gamma \log n$, since *C*(*r*) contains exactly $c_\varepsilon ^2 \gamma \log n$ **1**s and, by Lemma 4, fewer than $5c_\varepsilon \gamma \log n$ of them intersect $x_v$. So, the goal of our next lemma is to show that noise does not disrupt this by too much.

### Lemma 5

For sufficiently large constant $c_\varepsilon$, with probability at least $1-n^{4-c_\varepsilon \gamma }$, for all nodes *v*, $\tilde{R}_v = R_v$.

### Proof

Conditioning on the event of Lemma 4, all *C*(*r*) for $r\notin R_v$ $c_\varepsilon (c_\varepsilon - 5)\gamma \log n$-intersect $\lnot x_v$. Then, for such an *r* to be in $\tilde{R}_v$, more than ${\textbf {1}}(C(r) \wedge \lnot x_v)-\frac{2\varepsilon +1}{4}c_\varepsilon ^2\gamma \log n$ of the intersection positions would have to be flipped by noise. The probability of this is clearly minimized when ${\textbf {1}}(C(r) \wedge \lnot x_v)$ is as low as possible, i.e., $c_\varepsilon (c_\varepsilon - 5)\gamma \log n$. Then, $c_\varepsilon (c_\varepsilon - 5)\gamma \log n - \frac{2\varepsilon +1}{4}c_\varepsilon ^2\gamma \log n = (\frac{3-2\varepsilon }{4}c_\varepsilon - 5 )c_\varepsilon \gamma \log n$ positions must be flipped, and the expected number of such flipped positions is $\mu := \varepsilon (c_\varepsilon - 5)c_\varepsilon \gamma \log n$.

To show a low probability of failure, we need that the number of positions that must be flipped for *r* to be incorrectly categorized is more than its expectation. To do so, we bound the ratio of the two quantities:

$$\begin{aligned}&\frac{(\frac{3-2\varepsilon }{4}c_\varepsilon - 5 )c_\varepsilon \gamma \log n}{\varepsilon (c_\varepsilon - 5)c_\varepsilon \gamma \log n} = \frac{\frac{3-2\varepsilon }{4}c_\varepsilon - 5 }{\varepsilon (c_\varepsilon - 5)} \\  &\hspace{1in}\ge \frac{\frac{3-2\varepsilon }{4}c_\varepsilon - 5 }{\frac{c_\varepsilon }{2}}&\text { since } \varepsilon \in \bigg (0,\frac{1}{2}\bigg ) \\&\hspace{1in} =\frac{3}{2}-\varepsilon - \frac{10}{c_\varepsilon } \hspace{5.0pt}. \end{aligned}$$

We will set $c_\varepsilon \ge \frac{60}{1-2\varepsilon }$. Then,

$$\begin{aligned} \frac{(\frac{3-2\varepsilon }{4}c_\varepsilon - 5 )c_\varepsilon \gamma \log n}{\varepsilon (c_\varepsilon - 5)c_\varepsilon \gamma \log n}&\ge \frac{3}{2}-\varepsilon - \frac{1-2\varepsilon }{6} \\  &= \frac{4-2\varepsilon }{3}>1\hspace{5.0pt}. \end{aligned}$$

Now that we have bounded the ratio above 1, we can apply a Chernoff bound:

$$\begin{aligned}&\textbf{Pr}\left[ {\textbf {1}}(C(r) \wedge \lnot \tilde{x}_v) < \frac{2\varepsilon +1}{4} c_\varepsilon ^2\gamma \log n \right] \\  &\hspace{1cm}\le \textbf{Pr}\left[ \# \text { flipped positions } >\frac{\frac{3-2\varepsilon }{4}c_\varepsilon - 5}{\varepsilon (c_\varepsilon - 5)}\mu \right] \\&\hspace{1cm}\le exp(- \left( \frac{\frac{3-2\varepsilon }{4}c_\varepsilon - 5}{\varepsilon (c_\varepsilon - 5)}-1 \right) ^2 \mu /3 )\\&\hspace{1cm}\le exp(- \left( \frac{4-2\varepsilon }{3}-1 \right) ^2 \mu /3 )\\&\hspace{1cm}= exp(- \left( 1-2\varepsilon \right) ^2 \varepsilon (c_\varepsilon - 5)c_\varepsilon \gamma \log n/27 ) \hspace{5.0pt}. \end{aligned}$$

We will now further require that $c_\varepsilon \ge \frac{54}{\left( 1-2\varepsilon \right) ^2\varepsilon }+5$, which gives:

$$\begin{aligned}&\textbf{Pr}\left[ {\textbf {1}}(C(r) \wedge \lnot \tilde{x}_v) < \frac{2\varepsilon +1}{4} c_\varepsilon ^2\gamma \log n \right] \\  &\hspace{1cm}\le exp(- 2c_\varepsilon \gamma \log n) \le n^{-2c_\varepsilon \gamma } \hspace{5.0pt}. \end{aligned}$$

Conversely, for some $r'\in R_v$, *C*(*r*) does not 1-intersect $\lnot x_v$ (since it is contained in the superimposition that produces $x_v$). So, for it to $\frac{2\varepsilon +1}{4}c_\varepsilon ^2\gamma \log n$-intersect $\lnot \tilde{x}_v$, at least $\frac{2\varepsilon +1}{4}c_\varepsilon ^2\gamma \log n$ of the positions in which $C(r')$ has a **1** (of which there are exactly $c_\varepsilon ^2\gamma \log n$, by definition) would need to be flipped in $\tilde{x}_v$. The expected number of such flipped positions is $\mu ':=\varepsilon c_\varepsilon ^2\gamma \log n$. Since $\varepsilon \in (0,\frac{1}{2})$, have $\frac{2\varepsilon +1}{4}c_\varepsilon ^2\gamma \log n >\frac{4\varepsilon }{4}c_\varepsilon ^2\gamma \log n = \mu '$, so we can again apply a Chernoff bound:

$$\begin{aligned}&\textbf{Pr}\left[ {\textbf {1}}(C(r') \wedge \lnot \tilde{x}_v) \ge \frac{2\varepsilon +1}{4}c_\varepsilon ^2\gamma \log n \right] \\&\hspace{1cm}\le \textbf{Pr}\left[ \# C(r')'s {\textbf {1}}s \text { flipped } \ge \frac{2\varepsilon +1}{4}c_\varepsilon ^2\gamma \log n\right] \\&\hspace{1cm}\le exp(- \left( \frac{\frac{2\varepsilon +1}{4}}{\varepsilon }-1 \right) ^2 \mu '/3 ) \\&\hspace{1cm}=exp(- \left( \frac{1}{4\varepsilon }-\frac{1}{2} \right) ^2 \varepsilon c_\varepsilon ^2\gamma \log n/3 )\hspace{5.0pt}. \end{aligned}$$

Requiring that $c_\varepsilon \ge \frac{6}{\varepsilon }\left( \frac{1}{4\varepsilon }-\frac{1}{2} \right) ^{-2}$ again gives:

$$\begin{aligned}&\textbf{Pr}\left[ {\textbf {1}}(C(r') \wedge \lnot \tilde{x}_v) \ge \frac{2\varepsilon +1}{4}c_\varepsilon ^2\gamma \log n \right] \\  &\hspace{1cm}\le exp(-2c_\varepsilon \gamma \log n) \le n^{-2c_\varepsilon \gamma }\hspace{5.0pt}. \end{aligned}$$

So, each codeword is correctly placed in or out of $\tilde{R}_v$ with probability at least $1-n^{-2c_\varepsilon \gamma }$. Taking a union bound over all $2^{c_\varepsilon \gamma \log n}$ codewords, we have $\tilde{R}_v = R_v$ with probability at least $1-n^{-c_\varepsilon \gamma }$. Finally, taking another bound over all nodes $v\in V$ and removing the conditioning on the event of Lemma 4 (which occurs with probability at least $1- n^{3-c_\varepsilon \gamma }$ ) gives correct decoding at all nodes with probability at least $1-n^{4-c_\varepsilon \gamma }$. The lemma requires setting $c_\varepsilon \ge \max \{\frac{6}{\varepsilon }\left( \frac{1}{4\varepsilon }-\frac{1}{2} \right) ^{-2}, \frac{54}{\left( 1-2\varepsilon \right) ^2\varepsilon }+5,\frac{60}{1-2\varepsilon }\}$. $\square$

We now analyze the second stage of the algorithm, in which nodes transmit their messages using the combined code, and show that this code allows the messages to be decoded.

### Lemma 6

In the second phase of the algorithm, with probability at least $1-n^{\gamma +6-c_\varepsilon \gamma }$, all nodes *v* can successfully decode $\{m_w:w\in N(v)\}$ (so long as $c_\varepsilon$ is at least a sufficiently large constant).

### Proof

Conditioned on the event of Lemma 5, all nodes *v* now know $R_v$.

In the second stage of the algorithm, in the absence of noise *v* would hear the string $\bigvee _{w\in N(v)} CD(r_w,m_w)$, which we will denote $y_v$. To decode the message $m_w$, for some $w\in N(v)$, it examines the subsequence $y_{v,w}$ defined by $(y_{v,w})_j = (y_v)_i: {\textbf {1}}_j(C(w)) = i$. We denote the noisy versions of these strings that *v* actually hears by $\tilde{y}_v$ and $\tilde{y}_{v,w}$ respectively. (Note that *v* does not know which neighbor *w* the strings $r_w$ and $\tilde{y}_{v,w}$ belong to, but it can link them together, which is all that is required at this stage.) Node *v* decodes $m_w$ as the string $\tilde{m}_w\in \{0,1\}^{\gamma \log n}$ minimizing $d_H(D(\tilde{m}_w), \tilde{y}_{v,w})$. We must show that, with high probability, $\tilde{m}_w = m_w$.

Conditioned on the event of Lemma 4, each $C(r_w)$ for $w\in N(v)$ does not $5c_\varepsilon \gamma \log n$-intersect $\bigvee _{r_u\in R_v\setminus \{r_w\}} C(r_u)$. That is, there are at least $(c_\varepsilon -5)c_\varepsilon \gamma \log n$ positions in $x_v$ in which $C(r_w)$ has a **1** and no other $C(r_u)$ for $u\in N(v)$ does. In these positions *j*, $(y_{v,w})_j = D(m_w)_j$. So, in total,

$$\begin{aligned}d_H(D(m_w),y_{v,w}) \le 5c_\varepsilon \gamma \log n\hspace{5.0pt}.\end{aligned}$$

Under noise, each of the positions in which $y_{v,w}$ matches $D(m_w)$ will be flipped with probability $\varepsilon$ in $\tilde{y}_{v,w}$. So, denoting by $\mu$ the expectation $\textbf{E}\left[ d_H(D(m_w),\tilde{y}_{v,w})\right]$, we have:

$$\begin{aligned}\varepsilon c_\varepsilon ^2 \gamma \log n\le \mu \le \varepsilon c_\varepsilon ^2 \gamma \log n+ 5c_\varepsilon \gamma \log n \hspace{5.0pt}.\end{aligned}$$

Meanwhile,by the property of a $(\gamma \log n,\frac{1}{3})$-distance code, for any $m\ne m_w \in \{0,1\}^{\gamma \log n}$,

$$\begin{aligned}&d_H(D(m),y_{v,w}) \\&\hspace{1cm}\ge d_H(D(m),D(m_w))-d_H(D(m_w),y_{v,w})\\&\hspace{1cm}\ge \frac{1}{3} c_\varepsilon ^2\gamma \log n- 5c_\varepsilon \gamma \log n\hspace{5.0pt}. \end{aligned}$$

To lower-bound $\textbf{E}\left[ d_H(D(m),\tilde{y}_{v,w})\right]$ (which we denote by $\mu '$), we see that $\mu ' = (1-\varepsilon )d_H(D(m),y_{v,w}) + \varepsilon ( c_\varepsilon ^2 \gamma \log n- d_H(D(m),y_{v,w}))$. Since $\varepsilon >\frac{1}{2}$, this is minimized when $d_H(D(m),y_{v,w})$ is as small as possible, i.e., $\frac{1}{3} c_\varepsilon ^2\gamma \log n- 5c_\varepsilon \gamma \log n$. Then,

$$\begin{aligned} \mu '&\ge (1-\varepsilon )(\frac{1}{3} c_\varepsilon ^2\gamma \log n- 5c_\varepsilon \gamma \log n)\\&\hspace{1cm} + \varepsilon ( c_\varepsilon ^2 \gamma \log n- (\frac{1}{3} c_\varepsilon ^2\gamma \log n- 5c_\varepsilon \gamma \log n))\\&=(\frac{1}{3} c_\varepsilon - \frac{2}{3} \varepsilon c_\varepsilon - 5 + 10\varepsilon + \varepsilon c_\varepsilon )c_\varepsilon \gamma \log n\\&\ge \frac{1+\varepsilon }{3}c_\varepsilon ^2\gamma \log n -5c_\varepsilon \gamma \log n\hspace{5.0pt}. \end{aligned}$$

Since $\varepsilon <\frac{1}{2}$, we have $\frac{1+\varepsilon }{3}>\varepsilon$, and so we can see that for sufficiently large $c_\varepsilon$, $\mu \le \mu '$. So, it remains to show that $d_H(D(m_w),\tilde{y}_{v,w})$ and $d_H(D(m),\tilde{y}_{v,w})$ are concentrated around their expectations.

We first show that, with high probability, $d_H(D(m_w),\tilde{y}_{v,w}) \le \frac{1+4\varepsilon }{6}c_\varepsilon ^2\gamma \log n$. Note that if we set $c_\varepsilon \ge \frac{60}{1-2\varepsilon }$, then

$$\begin{aligned} \frac{1+4\varepsilon }{6}c_\varepsilon =\varepsilon c_\varepsilon + \frac{1-2\varepsilon }{6}c_\varepsilon > \varepsilon c_\varepsilon + 5 , \end{aligned}$$

and so

$$\begin{aligned}\frac{1+4\varepsilon }{6}c_\varepsilon ^2\gamma \log n> \varepsilon c_\varepsilon ^2\gamma \log n + 5c_\varepsilon \gamma \log n \ge \mu \hspace{5.0pt}.\end{aligned}$$

Then, we can apply a Chernoff bound:

$$\begin{aligned}&\textbf{Pr}\left[ d_H(D(m_w),\tilde{y}_{v,w}) \ge \frac{1+4\varepsilon }{6}c_\varepsilon ^2\gamma \log n \right] \\&\hspace{1cm}\le \textbf{Pr}\left[ d_H(D(m_w),\tilde{y}_{v,w}) \ge \mu \cdot \frac{(1+4\varepsilon )c_\varepsilon }{6( \varepsilon c_\varepsilon + 5)} \right] \\&\hspace{1cm}\le exp(- \left( \frac{(1+4\varepsilon )c_\varepsilon }{6\varepsilon c_\varepsilon + 30}-1 \right) ^2 \mu /2 ) \\&\hspace{1cm}= exp(- \left( \frac{(1-2\varepsilon )c_\varepsilon -30 }{6\varepsilon c_\varepsilon + 30}\right) ^2 \mu /2 ) \hspace{5.0pt}. \end{aligned}$$

The expression $\frac{(1-2\varepsilon )c_\varepsilon -30 }{6\varepsilon c_\varepsilon + 30}$ is increasing in $c_\varepsilon$. Therefore, if we ensure that $c_\varepsilon \ge \frac{30}{\varepsilon (1-2\varepsilon )}$, we have

$$\begin{aligned} \frac{(1-2\varepsilon )c_\varepsilon -30}{6\varepsilon c_\varepsilon + 30}&\ge \frac{\frac{30}{\varepsilon } -30}{\frac{180}{1-2\varepsilon }+ 30}\\  &= \frac{\frac{1}{\varepsilon } -1}{\frac{6}{1-2\varepsilon }+ 1}\\  &=\frac{(1-\varepsilon )(1-2\varepsilon ) }{\varepsilon (7-2\varepsilon )} \hspace{5.0pt}. \end{aligned}$$

Then,

$$\begin{aligned}&\textbf{Pr}\left[ d_H(D(m_w),\tilde{y}_{v,w}) \ge \frac{1+4\varepsilon }{6}c_\varepsilon ^2\gamma \log n \right] \\&\hspace{1cm}\le exp(- \left( \frac{(1-\varepsilon )(1-2\varepsilon ) }{\varepsilon (7-2\varepsilon )}\right) ^2 \mu /2 ) \\&\hspace{1cm}\le exp(- \left( \frac{(1-\varepsilon )(1-2\varepsilon ) }{\varepsilon (7-2\varepsilon )}\right) ^2 c_\varepsilon ^2\gamma \log n/2 )\hspace{5.0pt}. \end{aligned}$$

Finally, if we also ensure that $c_\varepsilon \ge 6\left( \frac{(1-\varepsilon )(1-2\varepsilon ) }{\varepsilon (7-2\varepsilon )}\right) ^{-2}$,

$$\begin{aligned}&\textbf{Pr}\left[ d_H(D(m_w),\tilde{y}_{v,w}) \ge \frac{1+4\varepsilon }{6}c_\varepsilon ^2\gamma \log n \right] \\&\hspace{1cm}\le exp(- 3c_\varepsilon \gamma \log n)\\&\hspace{1cm}\le n^{-4c_\varepsilon \gamma }\hspace{5.0pt}. \end{aligned}$$

We similarly wish to show that with high probability,

$$\begin{aligned}d_H(D(m),\tilde{y}_{v,w})> \frac{(1+4\varepsilon )}{6}c_\varepsilon ^2\gamma \log n\end{aligned}$$

(for $m\ne m_w$). Again, since we have set $c_\varepsilon \ge \frac{60}{1-2\varepsilon }$,

$$\begin{aligned}\frac{(1+4\varepsilon )}{6}c_\varepsilon = \frac{1+\varepsilon }{3}c_\varepsilon - \frac{1-2\varepsilon }{6}c_\varepsilon < \frac{1+\varepsilon }{3}c_\varepsilon -5\end{aligned}$$

So,

$$\begin{aligned}\frac{1+4\varepsilon }{6}c_\varepsilon ^2\gamma \log n< \frac{1+\varepsilon }{3} c_\varepsilon ^2\gamma \log n - 5c_\varepsilon \gamma \log n \le \mu '\hspace{5.0pt}.\end{aligned}$$

Then, we can apply a Chernoff bound:

$$\begin{aligned}&\textbf{Pr}\left[ d_H(D(m),\tilde{y}_{v,w})\le \frac{(1+4\varepsilon )}{6}c_\varepsilon ^2\gamma \log n\right] \\&\hspace{1cm}\le \textbf{Pr}\left[ d_H(D(m),\tilde{y}_{v,w}) \le \mu '\cdot \frac{(1+4\varepsilon )c_\varepsilon }{6(\frac{1+\varepsilon }{3}c_\varepsilon -5)} \right] \\&\hspace{1cm}\le exp(- \left( 1-\frac{(1+4\varepsilon )c_\varepsilon }{2(1+\varepsilon )c_\varepsilon - 30} \right) ^2 \mu /3 ) \\&\hspace{1cm}\le exp(- \left( \frac{(1-2\varepsilon )c_\varepsilon - 30 }{6\varepsilon c_\varepsilon + 30}\right) ^2 \mu /3 )\\&\hspace{1cm}\le exp(- \left( \frac{(1-\varepsilon )(1-2\varepsilon ) }{\varepsilon (7-2\varepsilon )}\right) ^2 c_\varepsilon ^2\gamma \log n/3 )\\&\hspace{1cm}\le exp(- 2c_\varepsilon \gamma \log n)\\&\hspace{1cm}\le n^{-2c_\varepsilon \gamma } \hspace{5.0pt}. \end{aligned}$$

Taking a union bound over all strings in $\{0,1\}^{\gamma \log n}$, we find that with probability at least $1-n^{\gamma -2c_\varepsilon \gamma }$, $d_H(D(m_w),\tilde{y}_{v,w}) < \frac{1+4\varepsilon }{6}c_\varepsilon ^2\gamma \log n$ and $d_H(D(m),\tilde{y}_{v,w})> \frac{(1+4\varepsilon )}{6}c_\varepsilon ^2\gamma \log n$ for all $m\ne m_w$. So, *v* successfully decodes $m_w$. Another union bound over all $w\in N(v)$ gives probability at least $1-n^{\gamma +1-2c_\varepsilon \gamma }$ that *v* correctly decodes the entire set $\{m_w:w\in N(v)\}$. Finally, removing the conditioning on the event of Lemma 5 and taking a further union bound over all nodes *v*, the probability that all nodes correctly decode their neighbors’ messages is at least $1-n^{\gamma +6-c_\varepsilon \gamma }$. We required that

$$\begin{aligned}c_\varepsilon \ge \max \left\{ \frac{30}{\varepsilon (1-2\varepsilon )}, 6\left( \frac{(1-\varepsilon )(1-2\varepsilon ) }{\varepsilon (7-2\varepsilon )}\right) ^{-2}\right\} \hspace{5.0pt}.\end{aligned}$$

$$\square $$

Lemma 6 shows that Algorithm 1 successfully simulates a Broadcast CONGEST communication round with high probability. By simulating all communication rounds in sequence, we can simulate any $n^{O(1)}$ Broadcast CONGEST in its entirety at an $O(\Delta \log n)$ overhead. Note that essentially all Broadcast CONGEST (and CONGEST) algorithms are $n^{O(1)}$-round, since this is sufficient to inform all nodes of the entire input graph. So the only problems with super-polynomial round complexities would be those in which nodes are given extra input of super-polynomial size. We are not aware of any such problems having been studied, and therefore Theorem 7 applies to all problems of interest.

### Theorem 7

Any $T=n^{O(1)}$-round Broadcast CONGEST algorithm can be simulated in the noisy beeping model in $O(T\Delta \log n)$ rounds, producing the same output with with probability at least $1-n^{-2}$.

### Proof

Each round of the Broadcast CONGEST algorithm, in which each node *v* broadcasts a $\gamma \log n$-bit message to all of its neighbors, is simulated using Algorithm 1 with sufficiently large constant $c_\varepsilon$. By Lemma 6, each simulated communication round succeeds (has all nodes correctly decode the messages of their neighbors) with probability at least $1-n^{\gamma +6-c_eps\gamma }$. Taking a union bound over all *T* rounds, and choosing $c_\varepsilon$ sufficiently large, gives a probability of at least $1-n^{-2}$ that all simulated communication rounds succeed. In this case, the algorithm runs identically as it does in Broadcast CONGEST, and produces the same output. The running time of Algorithm 1 is $O(\Delta \log n)$, so the overall running time is $O(T\Delta \log n)$. $\square$

We then reach an $O(\Delta ^2\log n)$-overhead simulation for CONGEST.

### Corollary 8

Any $T=n^{O(1)}$-round CONGEST algorithm can be simulated in the noisy beeping model in $O(T\Delta ^2 \log n)$ rounds, producing the same output with with probability at least $1-n^{-2}$.

### Proof

A $T=n^{O(1)}$-round CONGEST algorithm can be simulated in $O(T\Delta )$ rounds in Broadcast CONGEST as follows: nodes first broadcast their IDs to all neighbors, and then each CONGEST communication round is simulated in $\Delta$ Broadcast CONGEST rounds by having each node *v* broadcast $\langle ID_u, m_{v\rightarrow u}\rangle$ to its neighbors, for every $u\in N(v)$ in arbitrary order. Then, by Theorem 7, this algorithm can be simulated in $O(T\Delta ^2 \log n)$ rounds. $\square$

## Lower bounds

We now show lower bounds on the number of rounds necessary to simulate Broadcast CONGEST and CONGEST, based on the hardness of a simple problem we call *B*-bit Local Broadcast. We define the *B*-bit Local Broadcast problem as follows:

### Definition 5

(*B*-Bit Local Broadcast) Every node *v* is equipped with a unique identifier $ID_v\in [n]$. Every node *v* receives as input $\{\langle ID_u, m_{v\rightarrow u}\rangle : u\in N(v)\}$: that is, a set containing messages $m_{v\rightarrow u}\in \{0,1\}^B$ for each of *v*’s neighbors *u*, coupled with the ID of *u* to identify the destination node. Each node *v* must output the set $\{\langle ID_u, m_{u\rightarrow v}\rangle : u\in N(v)\}$ (i.e. the set of messages *from* each of its neighbors, coupled with their IDs).

### Lemma 9

*B*-Bit Local Broadcast requires $\Omega (\Delta ^2 B)$ rounds in the beeping model (even without noise), for any algorithm succeeding with probability more than $2^{-\frac{1}{2}\Delta ^2 B}$.

### Proof

The graph we use as our hard instance is as follows: we take the complete bipartite graph $K_{\Delta ,\Delta }$, and add $n-2\Delta$ isolated vertices. This graph then has *n* vertices and maximum degree $\Delta$. Arbitrarily fix unique IDs in [*n*] for each node. We will only consider the nodes of $K_{\Delta ,\Delta }$ to show hardness. Arbitrarily denote one part of the bipartition *L* and the other *R*. For nodes $v\in L$, we choose each $m_{v\rightarrow u}$ independently uniformly at random from $\{0,1\}^{B}$. We set all other $m_{x\rightarrow y}$ to ${\textbf {0}}^{\log n}$ (so, in particular, the inputs for all nodes $u\in R$ are identical).

Let $\mathcal {R}$ denote the concatenated strings of local randomness of all nodes in *R* (in any arbitrary fixed order). Then, the output of any node $u\in R$ must be fully deterministically dependent on the node IDs (which are fixed), $\mathcal {R}$, *u*’s input messages (which are identically fixed to be all ${\textbf {0}}$s), and the pattern of beeps and silence of nodes in *L* (and note that all nodes in *R* hear the same pattern: a beep if any node in *L* beeps, and silence otherwise). An algorithm running for *T* rounds has $2^T$ possible such patterns of beeps and silence.

So, the overall output of all nodes in *R* must be one of $2^T$ possible distributions, where the distribution is over the randomness of $\mathcal {R}$. The correct output for these nodes is uniformly distributed over $2^{\Delta ^2 B}$ possibilities (the choices of input messages for *L*). The probability of a correct output is therefore at most $2^{T-\Delta ^2 B}$. So, any algorithm with $T\le \frac{1}{2}\Delta ^2 B$ succeeds with probability at most $2^{-\frac{1}{2}\Delta ^2 B}$.

$$\square $$

Having shown a lower bound on the problem in the beeping model, upper bounds in Broadcast CONGEST and CONGEST imply lower bounds on the overhead of simulation.

### Lemma 10

*B*-Bit Local Broadcast can be solved deterministically in $O(\Delta \lceil B/\log n\rceil )$ rounds of Broadcast CONGEST and in $O(\lceil B/\log n\rceil )$ rounds of CONGEST.

### Proof

In Broadcast CONGEST, each node *v* simply broadcasts the strings $\langle ID_u, m_{v\rightarrow u}\rangle$ for each $u\in N(v)$, taking $O(\Delta \lceil B/\log n\rceil )$ rounds. In CONGEST, node *v* instead sends $m_{v\rightarrow u}$ to node *u* for each $u\in N(v)$, taking $O(\lceil B/\log n\rceil )$ rounds. $\square$

### Corollary 11

Any simulation of Broadcast CONGEST in the noiseless beeping model (and therefore also the noisy beeping model) has $\Omega (\Delta \log n)$ overhead. Any simulation of CONGEST in the noiseless (and noisy) beeping model has $\Omega (\Delta ^2 \log n)$ overhead.

## Applications

In this section, we list some of the applications of our result, simulating CONGEST and Broadcast CONGEST algorithms in the noisy beeping model.

### Degree computation

Exact node degrees can trivially be computed in 1 round in Broadcast CONGEST: each node broadcasts its ID, and nodes then count the number of IDs they received. Applying Theorem 7 therefore gives an $O(\Delta \log n)$-round algorithm for degree computation in noisy beeping networks:

#### Theorem 12

Degree computation can be performed in $O(\Delta \log n)$ rounds in the noisy beeping model, succeeding with high probability.

This improves significantly over the $O(\Delta ^2\log n + \log ^3 n)$-round algorithm obtained by applying the $O(\log n)$-overhead simulation result of Ashkenazi, Gelles, and Leshem [4] to the algorithm of [9] in the noiseless beeping model with collision detection.

### Distance-2 coloring

In vertex coloring, each node is required to output a color from some palette $\Phi$ such that no two adjacent nodes output the same color. It is a very well-studied problem in distributed computing, and the difficulty varies significantly depending on how many colors are in the palette. It is easy to see that a greedy coloring can always color using a palette of size $\Delta +1$, though in a distibuted setting this is non-trivial and colorings using more colors can be computed faster.

Distance-2 vertex coloring has the stronger requirement that nodes within distance 2, rather than 1, must output different colors. Equivalently, this can be thought of as coloring $G^2$, the square of the input graph. In this case, a greedy algorithm would require a palette size of at least $\Delta ^2+1$ colors. Distance-2 coloring is often used as a synchronization procedure in wireless networks, since it ensure that any node has at most one neighbor of any color class. For example, it is an integral part previous simulation results in the beeping model [4, 7].

The faster prior result was by Ashkenazi, Gelles, and Leshem [4], who gave a randomized algorithm for distance-2 coloring noisy beeping networks using $O(\Delta ^2)$ colors in $O(\Delta ^2\log n + \log ^2 n)$ rounds, based on simulating the algorithm of [9]. We improve this to $O(\Delta \log ^3 n)$, by simulating the following Broadcast CONGEST algorithm (Algorithm 2):

**Algorithm 2:**
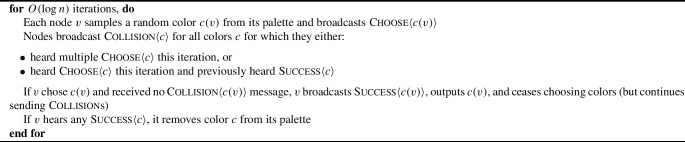
Distance-2 Coloring in Broadcast CONGEST

(Note that nodes are counted as *hearing* messages even if they were themselves the sender.)

#### Lemma 13

Algorithm 2 computes a $2\Delta ^2$ (list)-coloring of $G^2$ in $O(\log ^2 n)$ rounds of Broadcast CONGEST, succeeding with high probability.

#### Proof

Firstly, if the algorithm terminates, then it produces a correct $2\Delta ^2$ (list)-coloring of $G^2$. This is because whenever two nodes within distance 2 choose the same color *c* (either in the same or different iterations), each of their common neighbors (including themselves if they are in fact at distance 1) will broadcast $\textsc {Collision}\langle c\rangle$ and prevent the latter of the two (or both, if the choices were in the same iteration) from outputting *c*.

Next, we show that the algorithm terminates within $O(\log n)$ iterations with high probability. Each node has at most $\Delta ^2$ distance-2 neighbors ($\Delta$ at distance 1, and then a further $\Delta -1$ for each of those distance-1 neighbors). Therefore, for a fixed node *v*, in any iteration, at most $\Delta ^2$ total colors are either chosen by *v*’s distance-2 neighbors *this iteration* or have previously been output by such a distance-2 neighbor (since nodes that succeed and output a color then cease choosing colors). So, in any interation, *v* has a $\frac{1}{2}$ probability of choosing a color which has not been output or chosen by one of its distance-2 neighbors. In this case, *v* will hear no $\textsc {Collision}\langle c(v)\rangle$ messages and will successfully color itself. In expectation, therefore, at least $\frac{1}{2}$ of the remaining uncolored nodes will color themselves in each iteration. So, after $3\log n$ iterations, the expected number of remaining nodes is at most $\frac{1}{n^2}$, and so there are no remaining uncolored nodes with high probability by Markov’s inequality.

Finally, we show that we need only allow $3\log n$ rounds per iteration for broadcasting $\textsc {Collision}$s, with high probability. We analze the probability that *v* has more than $3\log n$ $\textsc {Collision}$s to broadcast in each iteration. If this is the case, then *v* must a set $S\subseteq {N(v) \cup \{v\}}$ of size $3\log n$, such that all of the colors chosen by nodes in *S* were unique, but all were also chosen or previously output by nodes in $N(v) \cup \{v\} \setminus S$. The number of choices for *S* is $$\left( {\begin{array}{c}\Delta +1\\ 3\log n\end{array}}\right) \le \Delta ^{3\log n}$$, and the probability that each member of *S* chose a color causing *v* to detect a collision is at most $\frac{\Delta }{2\Delta ^2} = \frac{1}{2\Delta }$, even conditioned on the choices of the other nodes in *S* since by definition they must have chosen a different color. So, the probability that node *v* has more than $3\log n$ $\textsc {Collision}$s to broadcast is at most $\Delta ^{3\log n} \cdot \left( \frac{1}{2\Delta } \right) ^{3\log n} \le n^{-3}$. Taking a union bound over all *n* nodes and $O(\log n)$ iterations gives the statement. $\square$

Applying Theorem 7 therefore gives a running time of $O(\Delta \log ^3 n)$ in the noisy beeping model:

#### Theorem 14

$2\Delta ^2$ (list)-coloring of $G^2$ can be performed in $O(\Delta \log ^3 n)$ rounds in the noisy beeping model, succeeding with high probability.

This improves the previous complexity by a factor of $\frac{\Delta }{\log ^2 n}$, a major improvement for large $\Delta$.

### Maximal matching

In this section we give another example application of our simulation, to the problem of maximal matching. The problem is as follows: we assume each node has a unique $O(\log n)$-bit ID. For a successful maximal matching, each node must either output the ID of another node, or Unmatched. The outputs must satisfy the following:

- Symmetry: *v* outputs *ID*(*u*) iff *u* outputs *ID*(*v*). Since each node outputs at most one ID, this implies that the output indeed forms a matching.
- Maximality: for every edge $\{u,v\}$ in the graph, *u* and *v* do not both output Unmatched.

To our knowledge, no bespoke maximal matching algorithm has previously been designed for the beeping model (either noisy or noiseless) or for Broadcast CONGEST. So, the fastest existing beeping algorithm is obtained by simulating the best CONGEST algorithms using the simulation of [4]. Since an $O(\Delta +\log ^* n)$-round CONGEST algorithm for maximal matching exists [29], the running time under [4]’s simulation is therefore $O(\Delta ^4\log n + \Delta ^3\log n \log ^* n )$.

We show an $O(\log n)$-round Broadcast CONGEST algorithm for maximal matching, which our simulation then converts to an $O(\Delta \log ^2 n)$-round algorithm in the noisy beeping model, thereby improving the running time by around a $\Delta ^3/\log n$ factor.

The base of our algorithm is Luby’s algorithm for maximal independent set [28], which can be applied to produce a maximal matching (Algorithm 3).^3^

**Algorithm 3:**
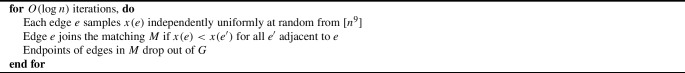
Maximal Matching: Luby’s Algorithm

It is well-known (see [28]) that Luby’s algorithm produces a maximal matching in $O(\log n)$ rounds with high probability. To implement this in Broadcast CONGEST we must make some minor changes to account for the fact that it is nodes, not edges, that communicate (Algorithm 4).

The aim of the algorithm is as follows: if, in a particular round *i*, an edge $\{u,v\}$ has a lower $x(\{u,v\})$ value than all neighboring edges, the following process occurs. Its higher-ID endpoint (assume W.L.O.G. that this is *u*) first broadcasts $\textsc {Propose}\langle \{u,v\}, x(\{u,v\})\rangle$. The other endpoint *v* then broadcasts $\textsc {Reply}\langle \{u,v\}\rangle$. Node *u* then broadcasts $\textsc {Confirm}\langle \{u,v\}\rangle$, and finally node *v* also broadcasts $\textsc {Confirm}\langle \{u,v\}\rangle$. These $\textsc {Confirm}$ messages cause nodes adjacent to *u* and *v* to be aware that *u* and *v* will be ceasing participation (because they have been matched), and so any edges to them can be discarded from the graph.

**Algorithm 4:**
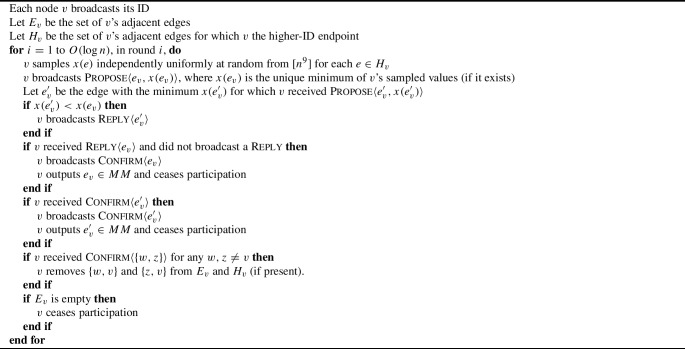
Maximal Matching in Broadcast CONGEST

#### Lemma 15

If Algorithm 4 terminates (i.e. causes all nodes to cease participation), it outputs a maximal matching.

#### Proof

We first prove maximality. Nodes only cease participation when they are adjacent to an edge in *MM*, or when they have no remaining adjacent edges. Edges are only removed when they are adjacent to an edge in *MM*. So, upon termination, there are no edges in the original graph that are neither in *MM* nor adjacent to an edge in *MM*, and therefore *MM* is a maximal matching.

We now prove independence. Let $\{u,v\}$ be an edge which is added to *MM* in round *i*, and assume W.L.O.G. that *u* is the higher-ID endpoint. It is clear that, since $\{u,v\}$ is added to *MM*, we must have the behavior described above (*u* broadcasts $\textsc {Propose}\langle \{u,v\}, x(\{u,v\})\rangle$, *v* broadcasts $\textsc {Reply}\langle \{u,v\}\rangle$, *u* broadcasts $\textsc {Confirm}\langle \{u,v\}\rangle$, *v* broadcasts $\textsc {Confirm}\langle \{u,v\}\rangle$). Then, we can show that this excludes the possibility that any adjacent edge also joins *MM* in round *i*:

1. *u* cannot act as the higher-ID endpoint of any other edge joining *MM*, since it only Proposes $\{u,v\}$.
2. *u* cannot act as the lower-ID endpoint of any other edge joining *MM*, since it Confirms an edge it Proposed, and therefore cannot have broadcast any Reply.
3. *v* cannot act as the higher-ID endpoint of any other edge joining *MM*, since it broadcasts a Reply and therefore does not $\textsc {Confirm}\langle e_v\rangle$.
4. *v* cannot act as the lower-ID endpoint of any other edge joining *MM*, since it only broadcasts Reply$\langle \{u,v\}\rangle$, and does not Reply for any other edge.

So, no adjacent edge to $\{u,v\}$ can join in round *i*. Furthermore, all nodes adjacent to *u* and *v* receive a $\textsc {Confirm}\langle \{u,v\}\rangle$ message and therefore all other edges adjacent to *u* and *v* are removed from the graph. So, no edge adjacent to $\{u,v\}$ can be added to *MM* in future rounds either. This guarantees that *MM* is an independent set of edges. $\square$

#### Notation 16

We will use the notation $e\sim e'$ to mean $e\cap e' \ne \emptyset$, i.e., $e'$ shares *at least* one endpoint with *e* (and can be *e* itself). We will denote $|\{e'\in E: e\sim e'\}|$ by *d*(*e*), i.e. the number of adjacent edges of *e*, including *e* itself.

#### Lemma 17

In any particular round *i*, the expected number of edges removed from the graph is at least $\frac{m}{2}$.

This lemma refers to the *current* graph at round *i*, i.e. without all edges and nodes that have been removed in previous rounds, and *m* is accordingly the number of edges in the current graph.

#### Proof

It is easy to see that, as intended, an edge $\{u,v\}$ is added to *MM* if it has a lower $x(\{u,v\})$ value than all neighboring edges: its higher-ID endpoint (W.L.O.G. *u*), which sampled the value $x(\{u,v\})$, will denote the edge as $e_u$ and Propose it, the $x(\{u,v\})$ value will be lower than that for which *v* Proposed and so *v* will $\textsc {Reply}\langle \{u,v\}\rangle$, and *u* will not hear a lower-valued edge to Reply and will therefore $\textsc {Confirm}\langle \{u,v\}\rangle$. *v* will also $\textsc {Confirm}\langle \{u,v\}\rangle$, and all edges adjacent to $\{u,v\}$ will be removed from the graph. There are $d(u)+d(v)-1$ such edges.

The probability that $x(\{u,v\})< x(e)$ for all $e\sim \{u,v\}$ with $e\ne \{u,v\}$ is $\frac{1}{d(u)+d(v)-1}$. So, the expected number of edges removed from the graph is at least $\frac{1}{2}\sum _{\{u,v\}\in E} (d(u)+d(v)-1)\frac{1}{d(u)+d(v)-1} = \frac{m}{2}$ (where the $\frac{1}{2}$ factor arises since each edge can be removed by either endpoint being matched, so is double-counted in the sum). $\square$

#### Lemma 18

Algorithm 4 performs maximal matching in $O(\log n)$ rounds of Broadcast CONGEST, succeeding with high probability.

#### Proof

By Lemma 15, Algorithm 4 produces a maximal matching if it terminates. Conditioning on the event that all sampled values are distinct, the algorithm removes at least half of the edges in the graph in each iteration in expectation. After $4\log n$ iterations, therefore, the expected number of edges remaining is at most $n^2 \cdot n^{-4} = n^{-2}$, and therefore by Markov’s inequaility, with probability at least $1-n^{-2}$ the number of edges remaining is 0 and the algorithm has terminated. Removing the conditioning on the event that sampled values are distinct, the algorithm terminates with probability at least $1-n^{-2}-n^{-4}$. $\square$

#### Theorem 19

Maximal matching can be performed in $O(\Delta \log ^2 n)$ rounds in the noisy beeping model, succeeding with high probability.

#### Proof

Follows from applying Theorem 7 to Lemma 18 . $\square$

This is close to optimal, since we show an $\Omega (\Delta \log n)$ bound even in the noiseless model:

#### Theorem 20

Maximal matching requires $\Omega (\Delta \log n)$ rounds in the (noiseless) beeping model, to succeed with any constant probability.

#### Proof

Our hard ensemble of instances is as follows: the underlying graph will be $K_{\Delta ,\Delta }$, the complete bipartite graph with $\Delta$ vertices in each part. Each node’s ID will be drawn independently at random from $[n^4]$.

Arbitrarily naming the two parts of the graph left and right, we consider the outputs of nodes on the right. For a correct output to maximal matching, each node on the right must uniquely output the ID of a node on the left, and so the union of outputs of the right part must be the list of IDs of the right part. The number of possible such lists (even assuming that IDs are all unique and the IDs of the right side are fixed) is $$\left( {\begin{array}{c}n^4-\Delta \\ \Delta \end{array}}\right) \ge \left( {\begin{array}{c}\frac{1}{2} n^4\\ \Delta \end{array}}\right) \ge \left( \frac{n^4}{2\Delta }\right) ^\Delta \ge n^{3\Delta }$$.

We note that each right node’s output must be dependent only on its ID, its local randomness, and the transcript of communication performed by left nodes during the course of the algorithm. Since the graph is a complete bipartite graph, in each round there are only two discernable possibilities for communication from the perspective of right-part nodes: either at least one left node beeps, or none do. So, the transcript for an *r*-round algorithm can be represented as a sequence $\{B,S\}^r$, corresponding to hearing a beep or silence in each round. There are $2^r$ such transcripts.

Therefore, the union of output from right nodes can be expressed as a function of the randomness of right-part nodes (both for their IDs and any local randomness used in the algorithm), and the transcript. Each transcript therefore induces a distribution of right-part outputs, over the randomness of right-part nodes.

There must be some set of left-part IDs such that under any transcript, the probability that the right-side nodes correctly output that set is at most $2^r / n^{3\Delta }$. So, if $r\le \Delta \log n$, then the probability that the right part produces a correct output on this instance is at most $n^\Delta / n^{3\Delta } = o(1)$. $\square$

### All-Pairs shortest paths

The All-Pairs Shortest Paths problem is another very well-studied graph problem, in which each node must output a minimum-distance path to every other node in the graph. The problem can be studied using either unweighted edges, in which case distance is standard graph distance, or with weighted edges, in which case the length of a path is the sum of all weights along that path.

#### Theorem 21

Unweighted All-Pairs Shortest Paths can be solved in $O(n\Delta \log n)$ rounds, and weighted All-Pairs Shortest Paths can be solved in $\tilde{O}(n\Delta ^2)$ rounds, in the noisy beeping model, succeeding with high probability.

#### Proof

For unweighted All-Pairs Shortest Paths, the *O*(*n*)-round algorithm of [24] can be simulated. This algorithm is written for CONGEST, but it can be seen that it in fact works unchanged in Broadcast CONGEST, and therefore can be simulated in $O(n\Delta \log n)$ rounds using Theorem 7. For weighted All-Pairs Shortest Paths, the $\tilde{O}(n)$-round CONGEST algorithm of Bernstein and Nanongkai [8] can be simulated in $\tilde{O}(n\Delta ^2)$ rounds. $\square$

## Conclusions

We have presented an optimal method for simulating Broadcast CONGEST and CONGEST in the noisy (and noiseless) beeping model. We have also presented several example applications, including a maximal matching algorithm which requires $O(\log n)$ rounds in Broadcast CONGEST, and which, using our simulation, can therefore be run in $O(\Delta \log ^2 n)$ rounds in the noisy beeping model.

While our general simulation method is optimal, there is still room for improvement for many specific problems in the beeping model, and the complexity picture has significant differences from the better-understood message passing models. For example, in CONGEST, the problems of maximal matching and maximal independent set have similar $O(\log \Delta + \log ^{O(1)}\log n)$ randomized round complexity upper bounds [5, 21, 30], whereas in the beeping model, maximal independent set can be solved in $\log ^{O(1)} n$ rounds [1] while maximal matching requires $\Omega (\Delta \log n)$ (Theorem 20). In general, the question of which problems can be solved in $O(\log ^{O(1)} n)$ rounds in the beeping model, and which require $poly(\Delta )$ factors, remains mostly open.

## Footnotes

1 Technically, *v* does not know which neighbor corresponds to which codeword, but this is not required by our approach.

2 For simplicity of notation, we will assume that a node counts a round in which it itself beeped as ‘hearing’ a ${\textbf {1}}$, and in the noisy model, flips this ${\textbf {1}}$ with probability $\varepsilon$ itself. Of course, in practice this is unnecessary, and having full information about its own message can only help a node.

3 Often this algorithm is stated with real values *x*(*e*) sampled from [0, 1]; however, since we must communicate these values using $O(\log n)$-bit messages, we instead use integers from $[n^9]$. It can be seen that with probability at least $1-n^{-4}$, no two of the values sampled during the algorithm are the same, so we can condition on this event for the rest of the analysis and avoid considering ties.
